# Teachers’ and Parents’ Assessments of Primary School Children’s Intellectual Investment as Predictors of Change in Need for Cognition

**DOI:** 10.3390/jintelligence13010010

**Published:** 2025-01-14

**Authors:** Anke Hufer-Thamm, Rolf Jürgens, Sebastian Bergold, Ricarda Steinmayr

**Affiliations:** Department of Psychology, TU Dortmund University, Emil-Figge-Straße 50, 44227 Dortmund, Germany; rolf.juergens@tu-dortmund.de (R.J.); sebastian.bergold@tu-dortmund.de (S.B.); ricarda.steinmayr@tu-dortmund.de (R.S.)

**Keywords:** need for cognition, elementary school, parental assessment, teacher assessment, investment traits

## Abstract

The present study investigated whether parents’ and teachers’ evaluations of children’s intellectual investment would predict a change in children’s need for cognition (NFC) over one year. An exploratory look at how teachers’ evaluations are predicted by a range of factors was also taken. *N* = 565 third-graders (298 girls; *M_age_* = 8.40, *SD* = 0.59) and teachers (*N* = 39) were surveyed in 2021 and 2022. The parents (*N* = 452) provided the data in 2021. Longitudinal data were analyzed by means of latent change score models (LCSMs). Changes in the teachers’ evaluations and in the children’s cognitive engagement differed between the children. However, there was no effect of the parents’ or teachers’ assessments on the development of the children’s NFC. The change in the teachers’ assessment was negatively related to their initial judgment and the children’s age; it was positively related to the pupils’ fluid intelligence. The results and implications are discussed.

## 1. Introduction

At the beginning of primary school, pupils have a strong motivation to learn (e.g., Jacobs et al. 2002), which usually declines in the following years (Spinath and Steinmayr 2008). This can have detrimental consequences for children’s future academic development, as motivation in elementary school predicts academic outcomes later in life (Musu-Gillette et al. 2015). Both teachers’ and parents’ judgments are known to influence children’s performance and cognitive development in various domains (e.g., Gudmundsson and Gretarsson 1994; Jussim and Eccles 1992; Korat 2004; Wigfield et al. 1999; Zippert and Ramani 2017). However, they do not only relate to the children’s achievement but also to their motivational development (Frome and Eccles 1998; Simpkins et al. 2015). Whereas the association between the assessment of children’s abilities and their skill development has been demonstrated for a whole range of cognitive abilities (e.g., Good 2024; Gudmundsson and Gretarsson 1994; Jussim and Eccles 1992; Jussim and Harber 2005; Korat 2004; Wigfield et al. 1999; Zippert and Ramani 2017), to our knowledge, there are no studies investigating this relation for investment traits, which describe how individuals deal with cognitively challenging situations (e.g., Mussel 2013). Such a connection would be important to better understand and support the motivational development of pupils. Some studies have addressed the motivational effects of ability assessments: Pomerantz and Dong (2006) showed that mothers’ assessments of their children’s ability affected their beliefs about the changeability of their own abilities, their preference for challenges, their self-esteem, and depression symptoms. High ratings were beneficial for the child, whereas low ratings tended to be detrimental. Teachers’ estimations of students’ competencies also seem to influence children’s school-related motivation and well-being (Bergold and Steinmayr 2023; Haimovitz et al. 2011; Hornstra et al. 2018; Steinmayr et al. 2019).

However, the extent to which parents’ and teachers’ perceptions of elementary school children’s motivation influence their motivational development and whether teachers and parents exert independent effects on children’s motivational development is still unclear for most motivational constructs and for investment traits. In the present study, we focus on the need for cognition (NFC), as it is highly relevant for educational outcomes (e.g., Strobel et al. 2024) and represents a central investment trait according to meta-analytical findings (von Stumm and Ackerman 2013). We investigated whether parents’ and teachers’ perceptions of children’s investment traits predicted the changes in the children’s NFC over the course of one year. Furthermore, we took an exploratory look at how the change in teachers’ assessments of children’s intellectual engagement is predicted by a range of control variables, including children’s fluid intelligence and parental education.

### 1.1. Investment Traits

Investment traits (e.g., Mussel 2010) are personality traits that affect how individuals deal with cognitive tasks, and they overlap considerably in theory and empiricism. Mussel (2013) organized the investment traits in his model according to two dimensions.

The first dimension in Mussel’s (2013) model is the *process* dimension and includes the two components *seek* and *conquer*. *Seek* investment traits motivate individuals to look for and begin cognitively challenging tasks, while *conquer* investment traits describe the effort that a person is prepared to invest in a current cognitive task. *Seek* thus describes the process that leads to a cognitive challenge, whereas *conquer* reflects how the investment trait affects the task’s processing.

The second dimension is *operation*, which consists of the three facets *think*, *learn*, and *create*. *Think* is related to fluid intelligence and describes how a person tends to behave in a problem-solving manner and enjoys or avoids complex situations. *Learn* relates to crystallized intelligence and reflects the desire to acquire new knowledge. People with low levels of *learn*, on the other hand, prefer to rely on their current skills and are less willing to expand their skills. *Create* refers to the tendency to become creative and develop new strategies, concepts, or unusual solutions.

### 1.2. Need for Cognition

Cohan et al. (1955, p. 291) defined NFC as “a need to structure relevant situations in meaningful, integrated ways. It is a need to understand and make reasonable the experiential world”. NFC is understood here as a need to reduce the inner tension that arises when there is a difference between current knowledge and the desired level of knowledge. According to Cacioppo et al. (1996), people with a higher NFC absorb more information from their environment in everyday life and make greater efforts to gain knowledge than individuals with lower levels. NFC can be analyzed within the Elaboration Likelihood Model as a tendency to choose central rather than peripheral processing of information (Cacioppo and Petty 1982; Petty and Cacioppo 1984). As a result, problems or facts, for example, are processed more deeply and receive more attention. As such, NFC is conceptualized as a personality trait, that is, as the general tendency to react to cognitively activating situations, as opposed to motivational variables that are more focused on specific goals or situations. In Mussel’s (2013) model, NFC is primarily assigned to the *seek* process and the *think* operation. NFC is thus primarily associated with seeking out cognitively challenging situations. In the hierarchical model proposed by von Stumm and Ackerman (2013), NFC is included in the first category that represents the core of investment (together with Typical Intellectual Engagement).

Empirically, NFC has been shown to be moderately associated with academic achievement (*r* = 0.20) in a recent meta-analysis and this relationship became stronger with an increasing grade level (Liu and Nesbit 2024). In addition, NFC has incremental validity with regard to academic achievement beyond intelligence and other predictors (e.g., Strobel et al. 2024). The tendency of people scoring high on NFC (“chronic cognizers”; Cacioppo et al. 1996, p. 197) to process information more profoundly (von Stumm and Ackerman 2013) and to exert more effort in problem-solving might lead to better academic performance. Due to both its conceptual relevance and its empirical association with academic achievement, we focus on NFC as a central investment trait in the present study.

### 1.3. Development of NFC in Young Age

Traditionally, NFC is seen as a relatively stable individual difference variable (Jebb et al. 2016), and a number of studies have found little or no difference between different age groups (e.g., Blanchard-Fields et al. 2001; Cacioppo et al. 1996; von Stumm 2012). However, there are only a few studies on the development of NFC overall, as it has almost exclusively been recorded as a predictor in the past. According to the results of Keller et al. (2019), NFC decreases from first to ninth grade (see also Luong et al. 2017, for grades three to nine). However, both studies were based on cross-sectional data. Preckel (2014) analyzed the development of NFC from the beginning of fifth to the end of sixth grade. These longitudinal results showed a decline in NFC over time.

With regard to rank-order stability, recent studies have reported stability coefficients of *r* = 0.56 in elementary school (Bergold and Steinmayr 2024; relying on the same data set as the current study), *r* = 0.50 between Grade 7 and Grade 11 (Lavrijsen et al. 2024), and *r* = 0.59 in late adolescence (Steinmayr et al. 2023). In a large meta-analysis, an average rank-order stability of *r* = 0.61 was shown for the Big Five Personality traits with an increasing stability throughout adolescence (Bleidorn et al. 2022). Thus, according to current knowledge, NFC appears to be comparably stable to the Big Five traits.

### 1.4. Socializers’ Impact on Change in NFC

Among others, the extended Situated Expectancy Value Theory (SEVT) (Eccles and Wigfield 2020) provides a theoretical framework for the development of children’s motivation. Although the SEVT does not focus on investment traits, its assumptions can be transferred to investment traits, and to NFC in particular. According to the SEVT, parents and teachers influence children’s expectancies and values such as intrinsic values, a construct that is theoretically different from but empirically similar to intrinsic motivation (Eccles 2005). As NFC is described as an intrinsic motivation to invest cognitive effort (Zerna et al. 2024), it can be assumed that its development is also affected by the behaviour of parents and teachers. In turn, parents and teachers are influenced by a number of factors, including their own characteristics (e.g., education and gender) and general beliefs (e.g., gender-role stereotypes). These factors affect both their assessment of the children and their behaviour towards them.

Investment theories (e.g., Ackerman 1996; Cattell 1987) state that individuals invest their fluid intelligence to accumulate crystallized abilities. However, they also suggest that people differ in their investment and that individuals with higher investment traits invest more. This behaviour might then foster intellectual development in the long term. Building on investment theories, the openness-fluid-crystallized-intelligence (OFCI) model by Ziegler et al. (2012) suggests two pathways between the development of openness as the central investment trait and cognitive development. According to the *environmental enrichment hypothesis*, high openness fosters the development of fluid intelligence (and the development of crystallized intelligence indirectly via fluid intelligence) because individuals high on openness seek out more novel and intellectually challenging situations. The *environmental success hypothesis* proposes that being more intelligent enables individuals to succeed in novel situations and to experience more success in cognitive challenges and thus to develop a higher enjoyment of such challenges, resulting in increasing investment traits.

The SEVT is a dynamic and reciprocal model, in which the expectations and values of children and their caregivers mutually influence each other. The perception and evaluation of their children lead the parents and teachers to adapt their behaviour (e.g., parenting style) and this affects the children’s outcomes (e.g., performance and motivation). If parents recognize and value their children’s high levels of investment traits (e.g., NFC), they will probably encourage participation in cognitively stimulating activities, such as reading books or playing chess. Particularly, reading to a child is associated with better academic achievement in the long run (OECD 2010). Furthermore, they might encourage them more to master a challenging task as they are convinced that their children like this. This increases the likelihood that the child will perceive such activities as valuable and be motivated to engage in them, which, in turn, also supports their cognitive development with a long-term impact. Indeed, parents who encourage their children to overcome challenges have a beneficial impact on the development of their children’s intelligence and motivation to learn, which, in turn, might lead to improved academic achievement (cf. Matthes and Stöger 2023). Teachers who recognize their pupil’s motivation to invest cognitive effort might give them more challenging tasks or engage in more frequent intellectual conversations with them, initiating similar processes as described for the parents. These supposed mechanisms are also in line with investment theory and with the environmental enrichment hypothesis by Ziegler et al. (2012). Thus, the development of children’s investment traits (e.g., NFC) should be positively associated with parents’ and teachers’ evaluations of children’s investment traits.

Furthermore, according to the SEVT model, children’s outcomes have an impact on parents’ (and teachers’) beliefs via the across-time path. Thus, children’s performance, NFC, and intelligence should also influence parents’ and teachers’ assumptions and judgments about the children. The reciprocal effect of children’s motivational characteristics on socializers’ evaluations has rarely been investigated (but see Kriegbaum et al. 2019).

### 1.5. Accuracy of Socializers’ Judgments

Teachers’ and parents’ impacts on children’s development might hinge on the accuracy of their judgments. For example, studies on teacher judgments have found that (mild) overestimations have positive effects on students’ academic and motivational development, whereas underestimations hamper student progress (e.g., Bergold and Steinmayr 2023; Kolovou et al. 2024; Meissel et al. 2022). However, recent studies have applied an advanced methodological approach to separate ratings’ effects reflecting just a positive evaluation from over- or underestimation effects. Their findings demonstrate that a positive evaluation, which is independent from the actual performance, probably yields a positive effect on academic achievement (Förster et al. 2022; Paschke et al. 2020, 2023). Thus, we also expected a positive effect from a positive socializers’ evaluation on NFC.

Judgment accuracy has three components (e.g., Urhahne and Wijnia 2021), two of which have been notably related to children’s outcomes: relative accuracy (i.e., the correlation between the judgment and characteristic) and absolute accuracy (i.e., the degree to which the judgment and characteristic match in terms of their level). As we inspected the correlations between judgments and children’s self-reported NFC, we focus on relative accuracy. Teachers’ judgments of students’ academic abilities have been found to correlate substantially with students’ actual abilities, although the correlations were far from perfect. Meta-analyses have revealed the strongest correlations for mathematical and verbal abilities (r ≈ 0.65; Südkamp et al. 2012), followed by intelligence (*r* = 0.50; Machts et al. 2016). The correlations with most motivational constructs were clearly lower, hovering around *r* = 0.20 (e.g., Steinmayr et al. 2019; Dicke et al. 2012; Spinath 2005).

Parents’ ratings of children’s math competencies yield incremental validity beyond teachers’ ratings and children’s objective performance and grades when predicting children’s ability in self-concepts in math (e.g., Steinmayr et al. 2019). This may be attributable to the observation that parents spend more time across a broader range of situations with their children compared to teachers, at least at primary school age. Thus, parental ratings capture some variance not accounted for by teacher ratings. Overall, parental and socializers’ judgment of children’s competencies and behaviours show medium associations (e.g., Steinmayr et al. 2019). Concerning parent’s judgment accuracy, most results deal with relative accuracy. Parental ratings of competencies and intelligence display a medium (intelligence) to high (mathematical competencies) correlation with actual abilities (Steinmayr and Spinath 2009; Steinmayr et al. 2019).

### 1.6. Present Study

Based on the SEVT (Eccles and Wigfield 2020), investment theory, and the OFCI model, it can be assumed that parents’ and teachers’ estimations of children’s investment traits influence their development. Since educators’ beliefs and behaviours affect children’s development (e.g., Good 2024; Harris et al. 1986), a high assessment should have a positive effect, and a low assessment should have a negative effect on the change in children’s motivation.

The present study examined whether the assessment of investment traits by parents (i.e., both mothers and fathers) and teachers relates to children’s development of NFC. If parents or teachers rate the child’s cognitive engagement highly, they might provide them with more learning opportunities that require elaborated thinking, which might in turn promote cognitive skills. Following the environmental success hypothesis, cognitive skills might in turn promote NFC. Therefore, we hypothesized that parents’ and teachers’ assessments of intellectual investment have a significant effect on the change in children’s self-reported NFC, with high assessments being supportive and low assessments being inhibitory. In addition, possible determinants of the change in teachers’ assessments were modelled as control variables, including children’s fluid intelligence and parents’ education, as these exert an influence on the expectations of the caregivers according to the SEVT (Eccles and Wigfield 2020). This approach aims to provide a broader perspective on teachers’ assessments, including their origins and effects. We also inspected teachers’ and parents’ relative judgment accuracy. Up until now, there are, to our knowledge, no studies on the accuracy of judgments about NFC. However, guided by previous findings on judgments of motivational variables, we expected correlations of *r* ≈ 0.20.

## 2. Materials and Methods

Prior to data analysis, we preregistered the study including the design, hypothesis, statistical models, and exploratory analyses within the Open Science Framework (OSF; https://osf.io/sp2b8, access date 14 December 2024).

### 2.1. Procedure and Participants

The data were collected as part of the authors’ project (Bergold and Steinmayr 2024). The first measurement time (t_1_) took place from August to October 2021. A total of *N* = 565 third-graders (298 girls, 261 boys, 6 with no gender specified; *M*_age_ = 8.40, *SD* = 0.59) in 52 classes from 22 schools in the Ruhr-Area in Germany participated. In the second measurement time (t_2_) in August and September 2022, *n* = 445 pupils (78.76% of the initial sample) took part, with a slightly higher dropout rate for pupils with lower intelligence (*d* = 0.33; see Bergold and Steinmayr 2024). Attrition was not systematically related to NFC scores.

Teachers and parents of the children were asked to also participate in the study. If possible, both mothers and fathers filled in the respective questionnaires. At t_1_, *N* = 452 parents (*n* = 283 mothers, *M_age_* = 38.76 years, *SD* = 5.14; *n* = 169 fathers, *M_age_* = 42.72 years, *SD* = 6.41) completed the parent questionnaire, and responses were available from at least one parent for *n* = 311 (55%) children. *In total*, 39 teachers (*M_age_* = 41.63 years, *SD* = 10.79) answered the teacher questionnaire at t_1_ and 34 teachers did so at t_2_.

No participants were removed from the data set. If it was apparent from the test protocols that the information provided by pupils could not be evaluated (e.g., misunderstandings in the test instructions), this information was coded as missing. One teacher at t_2_ (teaching 23 pupils) was not the same as at t_1_; therefore, the respective teacher assessment at t_2_ was coded as missing. We kept pupils who only participated in t_1_ in the sample to retain as much information as possible. The assessments at t_1_ and t_2_ took about 90 min and were conducted by trained student research assistants. Parents and teachers were given the survey documents and completed them at home or in school, respectively.

### 2.2. Children’s Questionnaire

Children’s NFC was measured using the scale developed by Preckel and Strobel (2017) for the assessment of NFC in primary school children. This scale is based on the NFC scale by Cacioppo and Petty (1982) and was successfully validated by Keller et al. (2019). It consists of 14 items (e.g., “I like doing tasks where I have to think a lot”), which were answered on child-appropriate Likert scales (different-sized stars) with four levels (1 = does not apply at all, 4 = fully applies). The NFC was measured at both time points. The internal consistency was α = 0.85/.87 (t_1_/t_2_) and ω = 0.86/.88 (t_1_/t_2_).

Fluid intelligence was assessed using the German short version of the Culture Fair Intelligence Test (CFT 20-R; Weiß 2006). This test consists of four subtests (series completion, classifications, matrices, and topological reasoning) with a total of 56 items (α = 0.77).

### 2.3. Parent and Teacher Questionnaire

The teachers answered the questionnaires at both measurement points (t_1_ and t_2_), while the parents only answered the questionnaire at t_1_.

Using a single-item question (i.e., “[The child] likes to deal with cognitively challenging tasks”), mothers, fathers, and teachers rated the extent to which the children tend to make intellectual investments on a 7-point Likert scale. Parents also indicated their school education and stated their highest school-leaving qualification.

### 2.4. Statistical Models

We used latent change score models (LCSMs) with NFC and the teachers’ evaluations of pupils’ tendency to take on intellectual challenges to examine the changes in those variables. For NFC, full scalar invariance has been demonstrated over time in the same data set in a recently published study (Bergold and Steinmayr 2024). To investigate the reciprocal effects stated in our hypotheses, we ran a bivariate LCSM. Here, latent NFC (modelled using a single indicator corrected for unreliability, i.e., internal consistency of NFC at each measurement point) predicts the latent change score of the teachers’ assessment and vice versa. Missing values were estimated using the full information likelihood approach.

**Figure 1:**
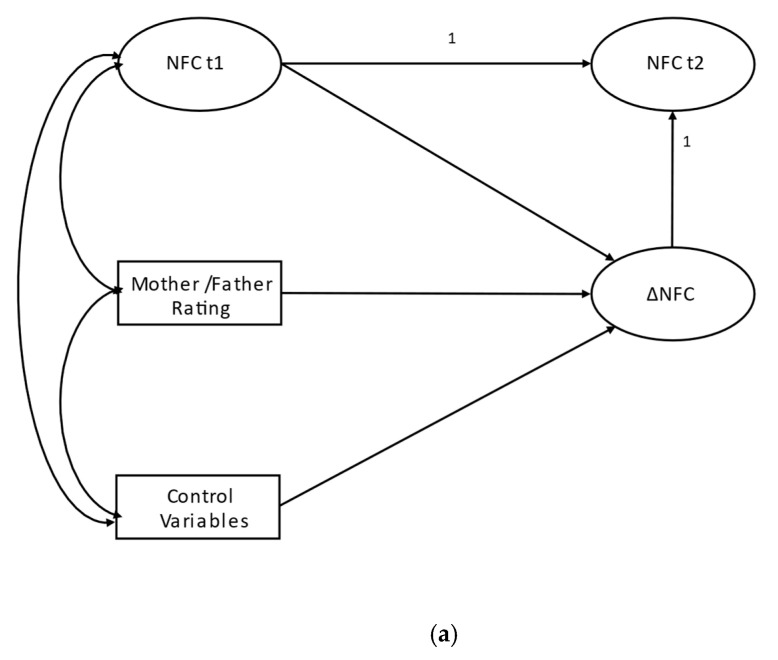

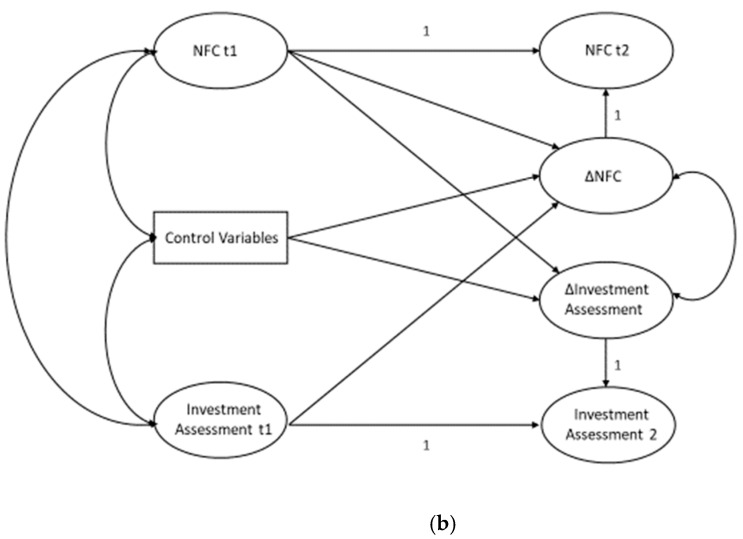
(**a**) Bivariate latent change model. Indicators of latent variables and correlations among corresponding indicators and among control variables are omitted for clarity. (**b**) Conditional latent change model. Indicators of latent variables and correlations among corresponding indicators as well as control variables are omitted for clarity.

As the parents were only asked to evaluate their children’s investment traits at t_1_, we conducted conditional LCSMs, in which the parents’ ratings predicted latent NFC at t_1_ and the latent change score. In order to avoid loss of information, we did not aggregate the parental assessments. Instead, mothers’ and fathers’ ratings were analyzed in separate models. Schematic depictions of the models are shown in Figure 1.

In the univariate LCSMs, we included age, gender, fluid intelligence, and parental education as control variables. For the bivariate analyses, we followed a two-step approach, in which we ran all models without control variables first and included the control variables in a second step. All variables included in the analyses were centered on the group mean (i.e., school classes). The LCSMs were calculated using Mplus 8.5 and all parameter estimates with a *p*-value below .05 were considered statistically significant. To account for the pupils being nested within classes, we corrected the standard error using the ‘type is complex’ command implemented in Mplus.

Additionally, we examined which variables predicted change in the teachers’ judgment of the pupils’ intellectual investment. For this purpose, the effects of NFC at t_1_ as well as the control variables (parental education, children’s fluid intelligence, age, and gender) on the change in teachers’ assessment from the bivariate LCSM were inspected. As a robustness check regarding the comparability of the NFC scale and the single item, we also conducted all bivariate analyses using the item with the highest similarity to the teachers’ and parents’ assessment (i.e., “I like doing tasks where I have to think a lot”).

## 3. Results

The descriptive values and correlations of the variables are presented in Table 1. The NFC values at t_1_ and t_2_ were highly correlated (*r* = 0.56, *p* < .001). The teachers’ assessments at t_1_ were significantly related to NFC at both t_1_ (*r* = 0.23, *p* < .001) and t_2_ (*r* = 0.28, *p* < .001). The teachers’ assessments at t_2_ were significantly connected to NFC at t_2_ (*r* = 0.29, *p* < .001). There was no significant connection between the NFC at t_1_ and the teachers’ ratings at t_2_. The mothers’ and fathers’ assessments of their children’s way of dealing with cognitive challenges were strongly correlated (*r* = 0.81, *p* < .001). All of the parent and teacher ratings were significantly but modestly interrelated, indicating only a small overlap between the assessment of the children’s intellectual investment behaviour shown in school vs. at home. In addition, the teachers’ ratings at t_1_ and t_2_ were very stable (*r* = 0.75, *p* < .001), even more so than the children’s NFC. Fluid intelligence was associated with all variables except for gender. It is notable that the correlations between the fluid intelligence and teachers’ ratings (*r*_t1_ = 0.51, *p* < .001; *r*_t2_ = 0.48, *p* < .001) were stronger than the correlations of intelligence with the parent ratings (*r*_mother_ = 0.21, *p* < .001; *r*_father_ = 0.32, *p* < .001). The associations between intelligence and NFC were small yet statistically significant (*r*_t1_ = *r*_t2_ = 0.16, *p* < .001) and the link between age and intelligence was slightly negative (*r* = −0.09, *p* = .044).

The results of the univariate LCSMs^1^ (see Table 2) show that neither the children’s NFC nor the teachers’ scores changed significantly on average, but the variances of change were significant for both variables. This implies that the change trajectories for NFC and teacher ratings differed among the children.

Table 3 shows the reciprocal relationships of the changes in the students’ NFC and in the teachers’ rating from the bivariate LCSM, with and without including the effects of the control variables. Only the NFC at t_1_ was a significant predictor of a change in NFC, with a medium negative association (β = −0.41, *p* < .001) with change (see also Bergold and Steinmayr 2024). As a result, NFC changed positively for children with low initial scores and tended to decrease for children with high starting levels.

Likewise, the first teacher assessment (t_1_) had a strong negative relationship with the change in the teacher’s rating (β = −0.64, *p* < .001), meaning that the children who were rated high at t_1_ were rated lower at t_2_ and vice versa. The pupils’ fluid intelligence had a slightly positive effect on the teachers’ ratings (β = 0.21, *p* < .001). A higher level of intelligence predicted stronger increases in the teachers’ ratings while a lower level of intelligence negatively predicted changes in ratings. The children’s ages had a weak negative effect on the change in the teachers’ ratings (β = −0.11, *p* = .027. The NFC at t_1_, parental education, and gender had no effect on the change in the teacher’s ratings.

The two separate conditional LCSMs for the mothers and fathers (see Table 4 with and Table 5 without the control variables) showed a small positive relation between the fathers’ ratings (t_1_) and their children’s NFC at t_1_, whereas the mothers’ ratings were not significantly related to NFC. Neither the mothers’ nor fathers’ ratings were significantly related to the changes in the children’s NFC. The results from the analyses using only one item from the NFC scale replicated the results from the main analyses.

## 4. Discussion

Whereas numerous studies have examined the impact of socializers’ ability ratings on performance outcomes (e.g., Good et al. 2018; Good 2024), and some have focused on motivational outcomes (e.g., Wang et al. 2018), little is known about how caregivers’ evaluation of *motivation* influences children’s motivational development. The present study thus investigated the relationship between the pupils’ NFC and the parents’ and teachers’ judgments of the pupils’ intellectual engagement in a sample of third-graders over the course of one year.

### 4.1. Correlational Findings

A closer look at the correlations between the study variables revealed that the teacher and parental ratings of the pupils’ way to deal with cognitive challenges had relatively weak associations, whereas the correlations between the mothers’ and fathers’ ratings were high. Differences between teachers’ and parents’ perceptions of children are a common finding and are often due to situational specificity (e.g., Bergold et al. 2019; De Los Reyes and Kazdin 2005). Therefore, our results might indicate that teachers and parents have very different perspectives in the sense that children might exert different intellectual investment behaviours. In school, teachers observe the children more frequently in cognitively challenging situations than parents do and they have other pupils as a comparison. Parents, on the other hand, may be able to see the child’s behaviour in situations in which the child has more possibilities to decide whether to engage in cognitive challenges or not. Thus, the specific situations might contribute to a different perception of students’ intellectual engagement.

Both of the other perspectives exhibited low to modest correlations with the children’s self-assessed NFC, which might show that the behaviour the child exerts does not necessarily align with their inner desire to engage in cognitively challenging endeavours. The found associations were mostly in line with what we expected based on previous research on motivational variables (e.g., Steinmayr et al. 2019). From a methodological view, the parents and teachers rated the pupils on only one item, while the children filled in a more nuanced measure, which could possibly lead to lowered correlations. Nevertheless, this does not invalidate our general findings, as the single item captures the core of intellectual investment and thus relates closely to NFC on a conceptual level (cf. von Stumm and Ackerman 2013). Furthermore, the teachers’ assessments showed a higher stability between the two measurement times than the self-assessed NFC even though it was measured with a single item. Thus, teachers do not seem to change their impression of a pupil’s handling of cognitive challenges much over the course of one year.

Most of the other correlations were in line with previous research, such as the relationships between the child’s intelligence and both maternal and paternal education (e.g., Cave et al. 2022). Likewise, the associations between NFC and fluid intelligence were comparable to findings from prior studies (e.g., Bergold et al. 2023; Scherrer et al. 2024).

### 4.2. Changes in NFC

The results showed that the self-reported NFC did not change on average between grade three and grade four (see also Bergold and Steinmayr 2024). Up until now, there have been only a few studies on the development of NFC in childhood or adolescence. Previous studies investigating different age cohorts suggested that there might be a decline from childhood to early adolescence (Keller et al. 2019; Luong et al. 2017; Preckel 2014). Interestingly, the difference between grade three and grade four in Keller et al.’s (2019) German samples was only marginal—like the decline in our sample. Scherrer et al. (2024) found no change in NFC from early to middle adolescence, whereas there seems to be an increase from late adolescence to early adulthood (Bergold et al. 2023; Bruinsma and Crutzen 2018). It might be that early adolescence is a turnaround in NFC development, in which the decreasing trend comes to an end and begins to turn into growth (see also Aerts et al. 2024). According to our and Keller et al.’s (2019) findings, the late elementary school years might be the starting point of this turnaround.

However, we found that the children differed in their change in NFC, which is consistent with findings from previous studies on the development of NFC (Steinmayr et al. 2023; Bruinsma and Crutzen 2018; Lavrijsen et al. 2024; Scherrer et al. 2024), motivational variables such as ability self-concepts (Jacobs et al. 2002), and investment traits other than NFC (Bergold and Steinmayr 2024). Given the relevance of NFC for academic achievement and well-being (Liu and Nesbit 2024; Zerna et al. 2024), detecting the source of variation in change is important.

### 4.3. Effects of Parent and Teacher Ratings of Intellectual Investment on Changes in NFC

Based on theoretical suppositions proposed by SEVT (Eccles and Wigfield 2020) and supported by investment theory and the OFCI model (Ziegler et al. 2012), we hypothesized that parental and teacher evaluations of the pupils’ tendency to engage in cognitively challenging activities would be positively related to changes in the children’s NFC. Our findings, however, hardly supported this assumption. Although we found significant associations between the parent or teacher ratings and the initial NFC level of the children, there was no significant relation between the parent or teacher ratings and change in NFC. How the parents or teachers rated the pupils’ tendency to enjoy cognitive challenges thus had no effect on how the children’s self-reported NFC changed over the span of one year.

These results differ from the effects of *performance* evaluation by the children’s socializers on the intellectual development of the pupils found in previous research (e.g., Good 2024). One reason for these differences could be that judgments by parents or teachers probably only have an effect on the child’s development if they manifest in some kind of behaviour which the child is actually able to perceive, such as providing more support to those perceived as highly capable. It is possible that assumptions about the child’s tendency to favour complex cognitive tasks do not translate as much into behaviour as ratings of the pupil’s performance.

Comparing our results to those from research on the effects of performance evaluation on *motivational* variables (e.g., Bergold and Steinmayr 2023; Rubie-Davies et al. 2006) can also offer insights regarding the outcome variables in question. For instance, Bergold and Steinmayr (2023) found effects of teachers’ ratings of mathematical and reading ability on ability self-concepts, but not on intrinsic motivation. This difference is noteworthy with regard to our study, as the ability self-concept has a pronounced cognitive component (i.e., assumptions about one’s academic capabilities and resulting expectations of success; e.g., Eccles and Wigfield 2002). By contrast, intrinsic motivation has a stronger affective component (i.e., inherent interest, enjoyment of the task itself; e.g., Ryan and Deci 2000). Thus, intrinsic motivation toward cognitive tasks might relate more closely to the concept of NFC than expectation constructs might. The (cognitive) judgments of caregivers might have a more distinctive effect on variables with a cognitive focus than on more affect-based constructs. If others consider the child to have high cognitive skills, this enhances the pupil’s self-concept, but it might not necessarily imply that the child also experiences higher enjoyment when faced with cognitive challenges. This having been said, it should be borne in mind that ability self-concepts and intrinsic motivation tend to be highly correlated, yet they are separable (e.g., Spinath and Steinmayr 2008).

Furthermore, based on the dynamic aspect of the SEVT (Eccles and Wigfield 2020), one might also assume that children’s NFC would impact the change in teachers’ perceptions. However, the results did not support this assumption. There are very few studies that have examined how student characteristics, such as motivation, affect teachers’ perceptions of students. Kriegbaum et al. (2019) demonstrated a reciprocal effect of students’ academic self-concept on teachers’ perception of students’ giftedness. However, they also focused on academic self-concept as a predominantly cognitive, rather than emotional, motivational variable. Similarly, just as in Bergold and Steinmayr (2023)), teachers’ perceptions of students’ performance did not affect changes in intrinsic motivation—an emotional–motivational variable—NFC also does not appear to be related to changes in teachers’ perception of students’ NFC. As we did not assess the parents’ estimations of their children’s NFC, we could not investigate the dynamic aspect of the SEVT (Eccles and Wigfield 2020) for parents’ estimations. Furthermore, the model depicts more complex effects of socializers’ beliefs on students’ motivational characteristics, such as an indirect effect of how students perceive their socializers’ estimations. As we did not assess most variables depicted in the SEVT, we were only able to investigate a very small part of the whole model.

### 4.4. Fluid Intelligence Related to Change in Teacher Ratings

An interesting result, apart from our hypothesis, was that fluid intelligence (included as a control variable) at the first measurement time was significantly related to changes in the teachers’ ratings of pupils’ cognitive engagement. The teachers’ judgment of their pupils’ intellectual investment increased more strongly for the smarter children than for the less intelligent children. Fluid intelligence is one of the strongest predictors of educational performance (e.g., Roth et al. 2015). Since teachers evaluate pupils based on their observed performance (Eccles and Wigfield 2020) and infer pupils’ motivation from this performance (Bergold and Steinmayr 2023), they may end up rating pupils’ intellectual investment higher. According to Cattell’s (1987) intelligence theory, the effect of fluid intelligence on academic achievement is mostly mediated via crystallized intelligence. Thus, it might be that the found effect would be even stronger if crystallized instead of fluid intelligence had been assessed. This notion is supported by studies demonstrating a higher correlation between crystallized (e.g., verbal) intelligence and academic achievement than that found for fluid intelligence (Roth et al. 2015).

The found effect could also be due to actual cognitive development, or might be due to teachers encouraging intellectual investment more strongly in pupils with greater aptitude. A potential moderator could be the teachers’ mindset (i.e., growth vs. fixed mindset; e.g., Yeager and Dweck 2012). The effect might be more prominent in teachers with a growth mindset (i.e., teachers who assume that cognitive abilities and engagement can develop). This hypothesis is worth investigating in future research. We were not able to evaluate the relationship between the assessments made by the teachers or parents and the children’s intelligence longitudinally based on our data.

Interestingly, the assessments made by teachers were found to be more strongly associated with the children’s intelligence than those made by parents (at t_1_). Teachers seem to base their evaluation of cognitive engagement more on cognitive ability than parents do. Consistent with previous research (e.g., Liu and Nesbit 2024; von Stumm and Ackerman 2013), the correlations between cognitive ability and NFC were low but significant in our sample (see also Bergold and Steinmayr 2024). There was, however, no association between fluid intelligence and the change in self-reported NFC.

### 4.5. Limitations and Future Directions

Despite the strengths of our study, some limitations need to be considered when interpreting the results. First, this was not an experimental but a correlational study, limiting conclusions about causal relations. Second, the assessments by teachers and parents were measured using a single item. This could be another reason for the relatively low correlations between the self-assessed NFC and the other ratings, even though the relations were mostly in line with those from previous research. While full scalar invariance was demonstrated for NFC (Bergold and Steinmayr 2024), it was not possible to test measurement invariance using only one item. Nevertheless, the single item captures the conceptual core of intellectual investment, comparably to NFC according to the model by von Stumm and Ackerman (2013). We thus think that the single-item measure does not diminish the overall validity of our results. In addition, a robustness check (i.e., running the bivariate analyses with the item from the NFC scale which was most similar to the single item answered by parents and teachers) revealed comparable results.

Third, we measured the parental judgments only at one measurement time, so that we could not depict changes in this variable. Both the teachers’ judgment and children’s NFC were measured at two time points, enabling us to model change in both the predictor and outcome. Nevertheless, regression to the mean could have been present, a statistical phenomenon where extreme measures tend to change toward their mean (Sorjonen et al. 2023) and which could have been one reason for the finding that NFC changed positively for children with low starting levels and tended to decrease for children with high initial values. In addition, two measurement points do not allow for the separation of relations within individuals from relations between individuals (e.g., Hamaker et al. 2015). Therefore, future research should include more than two measurement times. In this way, it would also be possible to model the development of NFC and the caregivers’ ratings over a longer period. Fourth, the systematic dropout of children with lower intelligence may have influenced the results, but did not lead to a change in variance for the individual variables and is low in strength (see Bergold and Steinmayr 2024). Fifth, we only investigated one investment trait, i.e., NFC. The results might differ if other investment traits are considered.

### 4.6. Implications and Conclusions

In the present study, we examined whether the teachers’ and parents’ ratings of primary school pupils’ tendency to engage in cognitively challenging tasks was associated with changes in the children’s self-reported NFC. Contrary to our hypothesis, derived from SEVT, investment theory, and the OFCI model, neither parental nor teacher assessments of pupils’ cognitive engagement were related to changes in the self-reported NFC. Interestingly, the fluid intelligence predicted changes in the teachers’ ratings of cognitive engagement.

These results can be useful for educational practice. It could be helpful if teachers were aware of the possibility that for the more intelligent pupils, their rating of the child’s cognitive engagement might change more positively. The potential underlying mechanisms and the behaviour towards the pupils can then be reflected or even be adapted. The correlational results draw the attention towards the differing perspectives of teachers and parents and to the implication that the child’s intellectual behaviour observed by others does not necessarily align with their inner desire to engage in cognitively challenging endeavours. From a theoretical point of view, our findings also provide a basis for further research with regard to investment traits in general and NFC in particular. At least according to our study, the suppositions drawn from the SEVT, investment theory, and the OFCI model do not seem to be transferable to investment traits (represented by NFC). As we were only able to analyze fractions of these models, our results may be a starting point for future research. Aerts et al. (2024) have recently proposed a theoretical framework for the development of NFC. With a focus on NFC, it might be worthwhile to see how our or upcoming findings can be incorporated into such broader frameworks.

## Figures and Tables

**Table 1:** Descriptive values and correlations.

	*M*	*SD*	Correlations									
			1	2	3	4	5	6	7	8	9	10
1 NFC t_1_	3.09	0.56	--									
2 NFC t_2_	3.03	0.55	0.56 ***	--								
3 Age	8.40	0.59	0.04	−0.00	--							
4 Gender	0.53	0.50	−0.01	−0.06	0.03	--						
5 Fluid intelligence	23.64	6.43	0.16 ***	0.16 ***	−0.09 ***	0.06	--					
6 Teacher’s rating t_1_	4.15	1.72	0.23 ***	0.28 ***	−0.22 ***	−0.15 **	0.51 ***	--				
7 Teacher’s rating t_2_	4.41	1.65	0.09	0.29 ***	−0.26 ***	−0.07	0.48 ***	0.75 ***	--			
8 Mother’s rating	4.37	1.24	0.14 *	0.11	−0.06	−0.05	0.21 ***	0.16 *	0.25 ***	--		
9 Father’s rating	4.51	1.31	0.20 *	0.04	−0.05	−0.09	0.32 ***	0.28 ***	0.31 ***	0.81 ***	--	
10 Education mother	3.52	1.31	0.00	0.08	−0.23 ***	−0.11	0.34 ***	0.36 ***	0.46 ***	0.14 *	0.15	--
11 Education father	3.56	1.29	0.06	0.24 ***	−0.23 ***	−0.01	0.25 ***	0.32 ***	0.30 ***	0.23 ***	0.24 **	0.54 ***

*Note.* NFC = need for cognition. Gender: 0 = male, 1 = female. *** *p* < .001, ** *p* < .01, * *p* < .05 (two-sided).

**Table 2:** Latent change in NFC and teachers’ ratings.

	Δ(SE)	σΔ(SE)
NFC	−0.01 (0.01)	0.12 (0.01) ***
Teacher rating	−0.08 (0.04)	0.80 (0.08) ***

*Note.* Unstandardized solution including control variables. NFC = need for cognition. *** *p* < .001

**Table 3:** Regression weights from the bivariate latent change score model with students’ NFC and teachers’ rating.

	ΔTeacher Rating	ΔNFC
Without control variables		
NFC t_1_	−0.00 (0.07)	−0.42 (0.06) ***
Teacher rating t_1_	−0.47 (0.05) ***	0.12 (0.06)
With control variables		
NFC t_1_	0.01 (0.07)	−0.41 (0.06) ***
Teacher rating t_1_	−0.64 (0.07) ***	0.05 (0.08)
Parental education	0.12 (0.07)	0.15 (0.09)
Fluid intelligence	0.21 (0.06) ***	0.00 (0.07)
Age	−0.11 (0.05) *	−0.03 (0.06)
Female gender	−0.04 (0.06)	−0.04 (0.06)

*Note.* Standard error in parentheses. *** *p* < .001, * *p* < .05.

**Table 4:** Conditional latent change score models with NFC and parent rating without control variables.

	NFC t_1_	ΔNFC
Mother’s rating t_1_	0.14 (0.09)	0.10 (0.06)
Father’s rating t_1_	0.23 (0.09) *	−0.19 (0.12)

*Note.* Standard error in parentheses. * *p* < .05.

**Table 5:** Conditional latent change score models with NFC and parent rating including control variables.

	NFC t_1_	ΔNFC
Mother’s rating t_1_	0.12 (0.09)	0.06 (0.07)
Father’s rating t_1_	0.20 (0.08) *	−0.19 (0.11)

*Note.* Standard error in parentheses. * *p* < .05.

## Data Availability

The data presented in this study are not publicly available. Interested researches my email the authors for insights into the data.

## Note

1 All models were just identified; thus, no fit indices are reported.
